# Astigmatism Management in Modern Cataract Surgery

**DOI:** 10.3390/vision8010009

**Published:** 2024-02-27

**Authors:** Royce B. Park, Ahmad A. Aref

**Affiliations:** Department of Ophthalmology, Illinois Eye and Ear Infirmary, University of Illinois at Chicago, Chicago, IL 60612, USA; rpark33@uic.edu

**Keywords:** astigmatism, cataract, surgery, phacoemulsification, toric, femtosecond

## Abstract

Astigmatism management is a frequently encountered challenge in the world of modern cataract surgery. This review article investigates the importance of astigmatic correction and seeks to uncover the critical components of preoperative evaluation. With the rapid growth of new technologies and techniques, this article aims to also catalogue and clarify the multitude of astigmatism treatment options available for the cataract surgeon.

## 1. Introduction

### 1.1. Prevalence

Globally, uncorrected refractive errors are a leading cause of vision impairment [1]. Of the different types, astigmatism is the most common refractive error in children and adults. The estimated pool prevalence of astigmatism across the world (40.4%) was found to be higher than myopia (26.5%) or hyperopia (30.9%) alone [2]. A recent study also estimated the prevalence of astigmatism at 57.4% and high astigmatism at 4.5% [3].

### 1.2. Age-Related Changes in Astigmatism

Changes in ocular astigmatism with age have been previously well-documented. Most eyes (55%) do not undergo astigmatic changes (in cylinder power or axis) until their mid-40s with many (41%) not experiencing a change until after their mid-50s [4]. This transformation is characteristically a conversion from with-the-rule (WTR) to against-the-rule (ATR) astigmatism, which studies have shown are three times more likely to rotate through oblique axes to return as ATR rather than reducing to zero and re-emerging as ATR [4]. Male eyes also exhibit greater and earlier ATR corneal astigmatism compared to female eyes, a sex-related difference with potentially significant implications for astigmatism correction [5].

Age-related astigmatic changes also demonstrably lead to a higher prevalence of anisometropia in older populations. In primary astigmatism, the prevalence of anisometropia increased from 7.6 to 17.8% over a 12-year period. In oblique astigmatism, it increased from 14.9 to 29.7% over the same period. Corneal, lenticular, and/or unequal axis changes in the cylinder component are thought to be contributory causes [6].

These age-related astigmatic trends are of particular importance as they pertains to cataract surgery, given the average age of this operative population. Of patients undergoing cataract surgery, over 40% were noted to have an astigmatism greater than 1D, and over 10% of those patients had astigmatism greater than 2D [7].

### 1.3. Economic and Fall Risk Impact

As with other refractive errors, uncorrected astigmatism poses a taxing burden on visual quality of life in terms of decreased acuity and increased visual disturbances. The economic toll on patients has also been documented, incurred through reduced education and employment opportunities and the need for often costly astigmatic correction. Most importantly, there is a significant decrease in health-related quality of life including difficulty with daily activities, driving impairment, and increased risk of falls [8]. It is estimated that a 1D degree of astigmatism can decrease visual acuity by up to 1.5 lines [9]. According to a recent study, uncorrected oblique astigmatism increases fall risk significantly more as compared to WTR and ATR astigmatism, further compounding the need for astigmatic management [10].

### 1.4. Anterior and Posterior Corneal Astigmatism

Astigmatism can be present on both the anterior and posterior surfaces of the cornea, which can be independent in magnitude and meridian from one another. Incorporating posterior cornea data in IOL calculation has been a matter of earlier contention [11]. Residual postoperative astigmatism after implantation of a toric IOL may be contributed to by posterior corneal astigmatism that was unaccounted for [12]. Previously, anterior corneal astigmatism (ACA) was measured directly by keratometers. Posterior corneal astigmatism (PCA) and total corneal astigmatism (TCA) were derived from an estimate using a keratometric index. Current technology has now permitted direct quantification of PCA values, thereby increasing the accuracy of TCA measurement [13]. Generally speaking, the relationship of posterior corneal astigmatism to overall refractive astigmatism is such that eyes with WTR ACA have a higher contribution from PCA (0.5–0.6D) than eyes with ATR ACA (0.2 to 0.3D) [12]. Additionally, PCA increases with higher WTR ACA [14]. Essentially, the posterior cornea is typically steeper along the vertical meridian in the grand majority of eyes, such that there is a greater impact to with-the-rule astigmatism [12].

### 1.5. Impact on Premium Intraocular Lens Outcomes

The implantation of a toric intraocular lens (IOL) is one of the most effective and common surgical methods to simultaneously treat cataracts and refractive astigmatism. However, residual astigmatism of more than 0.5D has been noted in up to 30% of postoperative eyes with toric IOL implantation [15]. Understandably, as residual astigmatism increases, uncorrected distance visual acuity worsens. Notably, the impact of multifocal IOL implantation on this relationship was found to be not statistically significant. Berdahl et al. [15] demonstrated that residual astigmatism affects visual acuity similarly in multifocal vs. monofocal toric IOL implantation.

## 2. Preoperative Evaluation

### 2.1. Calculation Methods/Formulas

Contributing factors to postoperative astigmatic “refractive surprise” include imperfections in preoperative measurement, corneal surgical-induced astigmatism, errors in alignment of the toric IOL, and the type of toric calculator used [16]. Huang et al. [16] compared outcomes between two calculators and found significantly lower postoperative astigmatism results when IOL toricity was determined by the Barrett Toric Calculator than by the AcrySof Toric Calculator. In the Barrett group, 89% of eyes achieved postoperative residual astigmatism less than 0.5D, compared to 53.7% of eyes in the AcrySof group. The Barrett Calculator’s incorporation of posterior corneal curvature and astigmatism likely minimizes error in the prediction of residual astigmatism [16].

A variety of recently developed toric IOL calculators are now available. The Barrett toric formula utilizes the Barrett Universal 2 formula to compute the effective lens position (ELP) and the posterior corneal astigmatism directly, or as a predicted value [17]. The Abulafia-Koch (AK) formula uses the Holladay 1 formula and the measured anterior corneal astigmatism to calculate the predicted refraction in each meridian [17]. The Naeser-Savini formula produces similar calculations using a combination of third-generation formulas. EVO 2.0 analyzes ELP and predicted posterior corneal astigmatism to calculate the total corneal power. The Holladay 2 formula includes correction for surgically induced astigmatism. The Kane toric formula calculates ELP and uses theoretical optics and artificial intelligence to estimate total corneal astigmatism [17].

Given the value of including PCA in preoperative measurements, Reitblat et al. [18] set out to investigate whether direct measurement of PCA was superior to estimation of PCA in terms of postoperative astigmatic outcomes. They compared the Barrett Toric Calculator using direct input of measured PCA against the Barrett Toric Calculator using predicted PCA, the Abulafia-Koch formula (which uses standard keratometry values to calculate total corneal astigmatism), and the Kane Toric formula. They found that direct input of measured posterior corneal astigmatism values resulted in similar outcomes for both the Barrett Toric Calculator (using predicted PCA) and the AK formula. Essentially, prediction errors were comparable between directly measuring PCA and estimating PCA.

### 2.2. Preoperative Ocular Surface Management

The impact of tear film stability on preoperative evaluation has also been studied. Work by Rochet et al. [19] revealed that instillation of artificial tears just prior to biometry and topography scans changed IOL cylinder calculation in 44% of eyes and shifted the implantation axis by more than 10 degrees in 18% of eyes. Eyes with increased dryness (a tear break up time of less than five seconds) demonstrated even higher rates of cylinder (58%) and axis (28%) change. Predicted error in astigmatism was significantly lower after instillation of artificial tears in these eyes.

### 2.3. Corneal Topography Limitations

The IOLMaster (Carl Zeiss Meditec, Dublin, CA, USA) has long been considered the gold standard for IOL measurements, using its automated keratometric readings [20]. With partial coherence interferometry, it derives the corneal power by measuring reflections of light on the cornea at six different points over a 2.3 mm radius. Such topographers, and manual or automated keratometers, are limited in their ability to measure beyond the anterior corneal surface. As a result, they do not fully capture the astigmatism (usually ATR) contributed by posterior corneal curvature [21]. Over one-third of ophthalmologists utilize topography automated biometry (IOLMaster/Lenstar) as the primary preoperative measure prior to toric IOL implantation, according to the most recent clinical survey by the American Society of Cataract and Refractive Surgeons (ASCRS) [22].

### 2.4. Corneal Tomography

**Figure 1:**
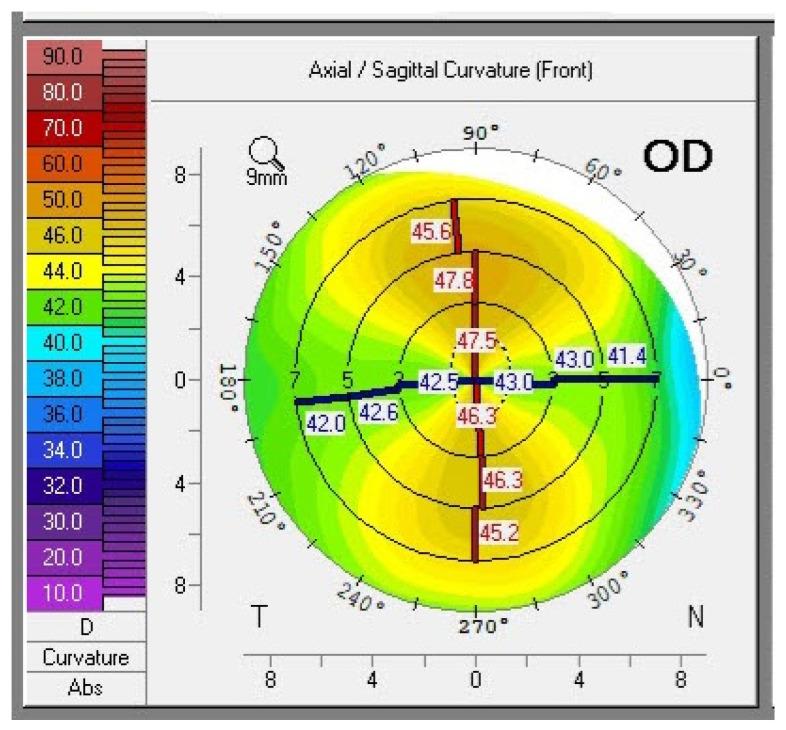
Corneal tomography printout indicating symmetric with-the-rule astigmatism.

In contrast, devices such as the Pentacam HR (Oculus, Wetzlar, Germany) utilize Scheimpflug technology to provide detailed renderings of the anterior and posterior corneal surfaces using up to 138,000 distinct elevation points (Figure 1) [20]. Thus, these devices can provide topography, tomography, pachymetry, keratometry, and anterior chamber photography. Seventeen percent of ASCRS survey respondents primarily favored Scheimpflug tomography (Pentacam/Galilei/Orbscan) [22].

### 2.5. Swept Source OCT

Swept-source optical coherence tomography (SS-OCT) is also a newer generation technology capable of measuring both the corneal front curvature and the back curvatures, in addition to intraocular distances and lens thickness [23]. The IOLMaster 700 (Carl Zeiss, Oberkochen, Germany) and the Argos (Movu, a Santec Company, Santa Clara, CA, USA) are two common SS-OCT biometers. They have demonstrated repeatability and reproducibility, in part due to the high-tissue penetration power of SS-OCT. Work by LaHood et al. [24] established the validity of total keratometry data obtained through the IOLMaster 700 by comparing it with the accepted Goggin nomogram [25] adjusted anterior keratometry (GNAK). Though just two percent of cataract surgeons favored OCT as their primary preoperative measurement technology, 30% of respondents based preoperative measurements on a varied combination of topography, tomography, OCT, autorefractor Ks, manual Ks, intraoperative aberrometry, and manifest astigmatism [22].

In comparing the predictive accuracy of measured PCA from the Pentacam HR versus the measured PCA from IOLMaster 700, Yang et al. [26] found no significant difference in residual astigmatism. The outcomes were comparable between the two technologies across the entire sample, WTR eyes, and ATR eyes, despite a significantly smaller posterior astigmatism measured by the IOLMaster 700. Athukorala et al. [27] also investigated SS-OCT keratometry (IOLMaster) against Scheimpflug images (Galilei G4 (Ziemer Ophthalmic Systems AG, Port, Switzerland)) and showed SS-OCT values more closely aligned with postoperative residual astigmatism.

Melendez et al. [28] then compared the IOL Master 700 to the Argos. Mean cylinder prediction error was not statistically significant between the two popular SS-OCT biometers. However, the spherical equivalent prediction error of 0.5D or less was significantly better with the Argos (80%) than the IOLMaster 700 (61%).

Table 1 [29] categorizes the different IOL biometers. It also details a non-exhaustive explanation of the general mechanisms, advantages, and disadvantages of each technology.

### 2.6. Posterior Corneal Astigmatism

The significant contribution of posterior corneal astigmatism to total astigmatism, and its impact on postoperative outcomes, has been well established. Sano et al. [30] showed that the difference between refractive and total corneal astigmatism (obtained based on both anterior and posterior corneal values) was less than the difference between refractive and keratometric astigmatism (obtained based on anterior corneal values alone), suggesting preoperative measurement of posterior corneal curvature could minimize unexpected postoperative astigmatism. Similarly, Srivannaboon et al. [11] identified analogous outcomes when using conventional keratometry versus total keratometry for IOL calculations, but found a trend toward lower refractive absolute errors when using total keratometry, which is inclusive of posterior corneal data.

Reitblat et al. [18] compared five different methods in calculating posterior corneal curvature for toric IOL implantation: (1) anterior corneal astigmatism using the Lenstar LS 900 (Haag-Streit, Koniz, Switzerland), a biometer with optical low-coherence reflectometry (OLCR), (2) the Baylor toric nomogram as described by Koch et al. [12], (3) vector summation from posterior tomography with the Scheimpflug camera plus anterior corneal astigmatism by the OLCR device, (4) true net power from the Scheimpflug camera, and (5) total corneal refractive power from the Scheimpflug camera. Of these five methodologies, performing vector analysis of posterior (Scheimpflug) and anterior (OLCR) corneal astigmatisms provided the lowest residual astigmatism results.

Corneal indices such as total corneal refractive power (TCRP), true net power (TNP), simulated keratometry (sim-K), and total keratometry (TK) can be utilized as variables in toric IOL measurements. The anterior and posterior curvature along with the refractive indices of the cornea and aqueous humor are incorporated with Snell’s law to calculate the TCRP using Scheimpflug technology [31]. TNP is based on a Gaussian formula involving the same variables as Scheimpflug technology. Simulated keratometry is based on the anterior corneal curvature and refractive index, which are used to estimate the total corneal power, assuming the cornea as a single refractive surface (constant corneal thickness and anterior to posterior curvature ratio), also using Scheimpflug technology [31,32]. TK is derived with measurement of both the anterior and posterior corneal curvatures using swept-source optical coherence tomography [32]. Studies have exhibited higher accuracy and reliability with TCRP as compared to TNP or sim-K [31]. By contrast, Shajari et al.’s [32] work demonstrated no significant difference between total keratometry compared to K-sim, TNP, and TCRP.

### 2.7. Surgically Induced Astigmatism

Clear corneal incisions are known to induce astigmatism iatrogenically due to flattening of the cornea in the meridian of the incision, resulting in a surgically induced astigmatism (SIA). This has led to the consideration of placing corneal wounds during phacoemulsification surgery at preoperative steep meridians. SIA is contingent not only on incision location, but also incision width, corneal diameter, surgical instrument used, and incision angle [33].

## 3. Correction of Low Astigmatism

### 3.1. Steepest Meridian Clear Corneal Incision

Steepest meridian clear corneal incisions can be utilized for correction of low astigmatism, typically 0.75 to 1.5D. Meridians can be marked preoperatively at the slit lamp, just prior to surgery in a fashion similar to marking for toric IOL implantation, in order to avoid the eye’s tendency to cyclorotate with supine positioning [34]. Using preoperative and postoperative measurements, each cataract surgeon can formulate a personalized SIA profile to predict the SIA effect, assuming routine and consistent corneal incisions by that individual surgeon [35]. However, published literature has reported a range of values in average SIA following clear corneal incisions, suggesting an unreliable predictability of surgically induced astigmatic changes. Langenbucher et al. [33] found that superior corneal incisions (12 o’clock) decreased astigmatism in the 90 degree meridian by around 0.25D. By contrast, Rho et al. [36] found superior corneal incisions resulted in an astigmatic decrease of 0.46D. Importantly, superior corneal incisions were noted to decrease keratometric astigmatism more than superotemporal (0.4D) or temporal incisions (0.28D). Hayashi et al. [34] reported changes ranging from 0.39 to 0.43D. Abulafia et al. [35] reported lower SIA values, ranging from 0.07 to 0.13D, with temporal incisions.

Opposite clear corneal incisions (OCCIs) have been described as a treatment for astigmatism in patients undergoing cataract surgery requiring up to 2D of astigmatism [37]. A second identical, paired, and opposite incision to the cataract CCI is made to enhance the flattening effect on the cornea.

### 3.2. Manual Corneal Relaxing Incisions

Limbal relaxing incisions (LRI) can be introduced at the time of phacoemulsification to correct pre-existing corneal astigmatism. The Nichamin nomogram [38] indicates the use of these arcuate incisions in WTR and ATR astigmatism ranging from 0.75 to 3.75D, though it is more commonly accepted in practice for correction up to 2D. Radial cuts are carried out at the limbus at a depth of 600 μm, extending from 30 to 90 degrees based on age, astigmatism axis, and astigmatism power. A second paired limbal relaxing incision is made 180 degrees away. Studies have demonstrated a significant reduction in topographic astigmatism that remained persistent through six months of postoperative follow-up [39]. Arcuate keratotomy (AK) is similar type of relaxing corneal incision that is placed more centrally (within 7–9 mm of the optical zone), imparting a greater change to corneal astigmatism [40].

### 3.3. Femtosecond Laser-Assisted Arcuate Keratotomy

**Figure 2:**
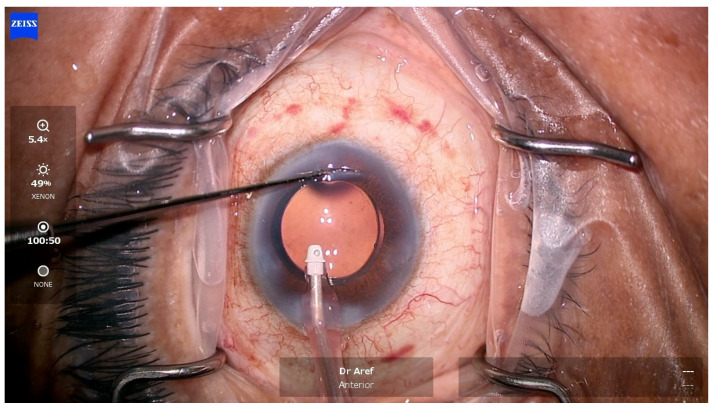
Intraoperative photograph demonstrating the opening of a femtosecond laser-assisted arcuate keratotomy. Note the location, length, and contour of this clear corneal arcuate incision nasally.

Femtosecond laser arcuate keratotomy (FLAK or FSAK) employs the assistance of a femtosecond laser to produce non-penetrating intrastromal corneal incisions in accordance with a nomogram as part of femtosecond laser assisted cataract surgery (FLACS) (Figure 2) [40].

The efficacy of femtosecond laser-assisted arcuate keratotomy is dependent upon arc length, arc depth, patient age, preoperative astigmatism power and axis, corneal biometric parameters, and the incision-to-limbus distance [7]. Studies by Zhang et al. [7] highlighted the incision-to-limbus distance as an independent predictor of surgically-induced astigmatism. They found that a greater incision-to-limbus distance resulted in increased astigmatism correction, even when the distance from the incision to the optical zone was the same. This was particularly relevant for populations with different corneal sizes, such as Asians with shorter corneal diameters [7], where the arcuate incisions were thereby placed closer to the limbus and the induced astigmatism magnitudes were much lower.

Schwarzenbacher et al. [41] found that reduction of anterior corneal astigmatism and total corneal astigmatism from femtosecond laser-assisted arcuate keratotomies persisted at 1-year follow-up. There was no significant change in posterior corneal astigmatism. Total corneal higher order aberrations also decreased significantly after femtosecond Aks.

### 3.4. Manual vs. Femtosecond Laser-Assisted Arcuate Keratotomy

González-Cruces et al. [40] performed a systematic review comparing manual relaxing incisions with FLAK and found no significant difference in uncorrected distance visual acuity or residual astigmatism. There was also no significant difference in refractive stability after three months.

Though serious complications are overall rare with manual incisions, there is a theoretical greater risk of perforation, wound leaking, epithelial downgrowth, keratitis, and endophthalmitis. These complications are seemingly less likely with the femtosecond laser arcuate keratotomy procedure [40]. Gas breakthrough, incorrect placement of AK incisions, and endothelial damage have been reported infrequently with FLAK. Femtosecond laser-assisted arcuate keratotomy is inclusive of both trans-epithelial (penetrating) and intrastromal (non-penetrating) procedures. Trans-epithelial arcuate incisions are placed at up to a depth of 85%, whereas intrastromal incisions involve the central 60% and preserve the upper 20% [42]. Some argue that this may lead to increased safety by avoiding epithelial hyperplasia and anterior stromal inflammation [42]. When comparing manual vs. femtosecond laser-assisted arcuate keratometry, since the refractive outcomes are statistically similar and the unique risks are low in both groups, the greater economic cost must be weighed against the greater precision and reproducibility of FLAK [40].

### 3.5. Arcuate Keratotomy vs. Toric IOL Implantation

Work by Yoo et al. [43] showed no statistically significant difference in refractive astigmatism outcomes between femtosecond laser assisted cataract surgery with trans-epithelial arcuate keratotomy versus conventional cataract surgery with toric IOL implantation. Their later work [42] also technically demonstrated no statistically significant difference between FLACS with intrastromal AK versus conventional cataract surgery with toric IOL implantation. However, results did trend toward lower postoperative refractive astigmatism with toric IOL placement. Patients in both groups had low astigmatism preoperatively, ranging from 0.75 to 2D, and were followed up to six months.

### 3.6. Toric Intraocular Lens

Toric IOLs were originally designed for correcting moderate-to-high degrees of astigmatism and remain a gold standard in the field. However, low-powered toric intraocular lenses (cylinder power of +1D) can be successfully implanted in eyes with corneal astigmatism as low as 0.5D [44]. Studies have demonstrated that the refractive outcomes of these eyes are better than those receiving only spherical IOLs. Recent work by Heinert et al. [45] illustrated that toric IOLs can effectively reduce low astigmatism (ranging from 0.75 to 1.5D) and significantly improve visual acuity as compared to non-toric IOLs. However, given the premium cost of toric IOLs, a cost-benefit analysis of low-powered toric IOL implantation remains to be further explored [46].

### 3.7. Light Adjustable Lens

A light adjustable lens (LAL) (RxSight Inc., Aliso Viejo, CA, USA) is a three-piece silicone lens implanted in the posterior chamber that has the capacity to be modified postoperatively. Controlled application of 365 nm ultraviolet can active photosensitive molecules, known as macromers, and adjust the spherocylindrical power in 0.25D increments [47]. Postoperative adjustment sessions are scheduled weeks after initial cataract surgery, whereupon the final spherocylindrical power can be “locked-in” when the desired refraction is achieved. These lenses are indicated for spherical adjustment from −2 to +2D, and cylindrical adjustment from −0.75 to −2D, providing a unique opportunity to non-invasively overcome residual refractive error [48]. Observed complications were similar to the monofocal control group, and no increased surgical risk was perceived as compared to conventional cataract surgery [48]. Moshirfar et al.’s [48] study showed 71% of patients achieved astigmatic correction of 0.5D or less 12 months postoperatively.

## 4. Correction of Moderate-to-High Astigmatism

### 4.1. Toric Intraocular Lens

Toric IOL implantation continues to be the procedure of choice in the correction of moderate or high astigmatism ranging from 1.25 to 3D. Hernandez et al. [9] compared toric IOL implantation with FSAK in cases of moderate astigmatism and found improved visual acuity and residual spherical equivalence with the toric group. Toric IOLs offer excellent predictability (minimal residual astigmatism not privy to corneal healing patterns), safety (relative to risks involved with corneal relaxing incisions), and long-lasting stability.

### 4.2. Intraoperative Alignment Technology

**Figure 3:**
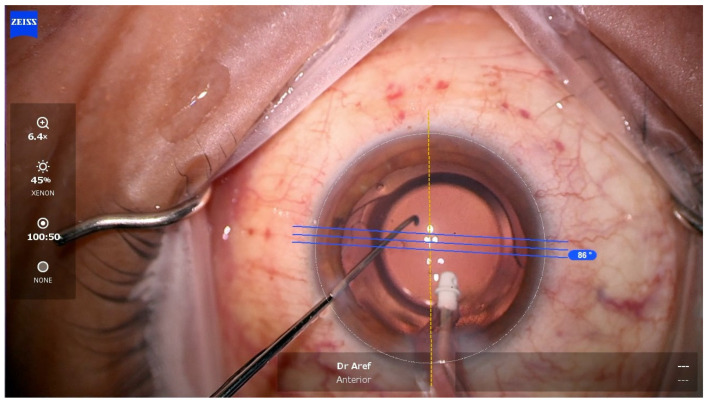
Intraoperative photograph demonstrating use of operating microscope overlay tool for alignment of a toric intraocular lens.

Marking of the corneal horizontal axis (0 and 180 degrees) in a seated position with a surgical marker is a standard preoperative procedure prior to toric IOL implantation. The Callisto (Carl Zeiss Meditec, Dublin, CA, USA) software (version 3.7) automatically matches the conjunctival limbal vessels from the preoperative biometry visit to the supine intraoperative eye under the microscope (Figure 3) [49]. This takes into account the natural cyclotorsion of the eye. Marking the eye for eventual toric IOL alignment is a critical step that demands precision. It has been well-established that misalignment of a properly powered toric IOL by 10 degrees will reduce its efficacy by 30%. Misalignment by more than 30 degrees will actually worsen the astigmatism in a different axis. Raucau et al. [49] compared manual versus automated horizontal axis marking in a single surgeon study and found that the two methods differed by an average of only 4.7 degrees. Yet, half of the 50 cases studied had a difference greater than 5 degrees. This degree of misalignment can significantly deteriorate refractive outcomes in cases where high-powered toric IOLs are used [49]. Comparatively, Kose et al. [50] compared refractive outcomes in eyes aligned with manual marking versus eyes aligned with the automated Callisto software and found no significant differences in visual acuity or residual astigmatism between the two groups. Overall, there was less toric misalignment in the automated group, but this did not have an impact on visual acuity at three months of follow-up.

### 4.3. Intraoperative Aberrometry

Intraoperative aberrometers (IA), such as the Optiwave refractive analysis system (ORA) (Alcon Laboratories, Inc., Fort Worth, TX, USA), attach to a surgical microscope and obtain refractive measurements intraoperatively in order to further supplement IOL selection [51]. Some studies [51,52] have demonstrated moderate improvement in refractive outcomes when using ORA versus conventional preoperative planning alone while others still [53] have elicited no significant difference. According to work by Davison et al. [54], no statistically significant difference in residual astigmatism using IA versus the Barrett toric calculator for toric IOL implantation was found.

## 5. Postoperative Residual Astigmatism

### 5.1. Prevalence and Etiology

Residual astigmatism postoperatively can be a primary source of poorly optimized vision and dissatisfaction among patients [40]. Significant astigmatism after toric IOL implantation can be quite common. Some studies have reported 28% of eyes with residual astigmatism greater than 0.5D, while others have cited a rate of 47% [55]. Postoperative astigmatism has been noted to range from 0.00 to 2.25D. Residual astigmatism results from incorrect axial orientation and/or from inaccurate cylinder power.

As mentioned in the sections and cited works prior, variables responsible for residual astigmatism include posterior corneal astigmatism, surface tear film stability, surgically-induced astigmatism, and toric IOL calculation. Kramer et al. [55] also found that residual refractive astigmatism tended to be higher in eyes with higher cylinder power IOLs. Investigative work by Hirnschall et al. [56] analyzed primary sources of error. Most common were preoperative measurement of the cornea (27%), IOL misalignment (14%), and IOL tilt (11%).

Additionally, astigmatism continues to advance as the cornea ages. Hayashi et al. [34] showed an ATR change of 0.2–0.4D over 10 years. This phenomenon of ATR astigmatic shift occurs irrespective of the native type of astigmatism and the age of the patient at time of cataract surgery, and appears to develop in both phakic and pseudophakic eyes alike [57,58].

### 5.2. Management

In cases of symptomatic cylindrical refractive error that persists after cataract surgery, management options include limbal relaxing incisions and IOL repositioning. LRIs can be performed manually or with the assistance of a femtosecond laser. Toric IOLs can be surgically rotated and repositioned along their ideal axis with the assistance of specific calculation tools. An appropriate length of time should pass to allow the cornea to heal and the capsule to contract, permitting a more accurate refraction to be established [59]. In the case of LRIs, six weeks postoperatively or more is recommended. Oshika et al. [60] analyzed toric IOL repositioning outcomes and concluded that misalignment should be corrected no earlier than one week postoperatively. The IOL tended to re-rotate in the capsular bag significantly up until the first postoperative week. IOLs repositioned at three weeks postoperatively or later were difficult to rotate and prone to damaging the zonules.

Studies have illuminated differences between toric IOLs in susceptibility to intraocular rotation and subsequent need for repositioning. Kramer et al. [61] found that of 6482 eyes implanted with either a TECNIS (Johnson & Johnson Vision, Jacksonville, FL, USA) or AcrySof (Alcon Laboratories, Inc., Fort Worth, TX, USA) monofocal toric IOL, 1.3% of eyes underwent surgical IOL repositioning. Repositioning incidence was significantly higher with TECNIS (3.1%) than AcrySoft IOLs (0.6%), with an odds ratio of 5.6. Younger age was also a significant risk factor for IOL repositioning. This was thought to be secondary to younger patients possessing higher visual expectations, increased postoperative physical activity inducing rotation, and different biomechanics of the capsular bag and toric axis (WTR vs. ATR) [61].

As mentioned previously, newer lenses have the ability to be modified postoperatively, thereby theoretically optimizing postoperative refraction. This dynamic process occurs through digital light (UV light) delivery. Chayet et al. [62] demonstrated a successful reduction of toric error in patients with postoperative astigmatic refractive errors ranging from 1.25–1.75D. Refractions remained stable through to the nine month follow-up.

Surface ablation procedures, such as laser-assisted in situ keratomileusis (LASIK), can be utilized to manage postoperative residual astigmatism. Norouzi et al. [63] found that LASIK was effective, predictable, and safe in correcting astigmatism ranging from 3 to 6D when performed one year after routine cataract surgery.

Lastly, postoperative residual astigmatism can be managed non-surgically through the use of reliable spectacles and contact lens wear, depending on cost, preference, lifestyle, comfort, corneal health, and degree of astigmatism.

## 6. Conclusions

Astigmatism is a common and critical component of optimal refractive outcomes after cataract surgery. Residual refraction can significantly reduce visual acuity and patient satisfaction, especially in today’s context of higher patient expectations with modern cataract surgery. Preoperative management should incorporate tear film stability, posterior and anterior corneal data, and validated toric calculators. Surgical management is quite varied, and ranges from manual corneal incisions to laser-assisted keratotomies to toric IOL implantation and LAL implantation. The development of, and increasingly widespread access to, assistive technologies such as femtosecond laser, intraoperative alignment, and intraoperative aberrometry have augmented the precision capabilities of cataract surgeons. Ultimately, the choice of technique in treating refractive astigmatism is dependent upon surgeon experience, the magnitude and axis of astigmatism, biometry data, cost, and equipment availability.

## Figures and Tables

**Table 1:** IOL biometers categorized by technology.

Technology	PCI/OLCI/OLCR	Scheimpflug	SS-OCT
Instruments	IOLMaster 500 AL-Scan OA-1000 Lenstar LS900 Aladdin	Pentacam HR Galilei G4	IOLMaster 700 Argos OA-2000 ANTERION
Mechanism	Low or partial-coherence Interferometry with topography	Three-dimensional rendering of the anterior segment	High-resolution cross-sectional images of the eye
Advantages	Efficiency, accuracy	Detailed rendering, irregular corneas	Resolution, accuracy
Disadvantages	Limited parameters, limited detail or resolution	Cost, proper alignment	Cost

PCI—partial coherence intereferometry; OLCI—optical low coherence interferometry; OLCR—optical low coherence reflectometry; SS-OCT—swept source optical coherence tomography; IOLMaster 500 (Carl Zeiss Meditec, Dublin, CA, USA); AL-Scan (Nidek Co., Ltd., Gamagori, Japan); OA-1000 (Tomey, Nagoya, Japan); Lenstar LS900 (Haag-Streit, Koniz, Switzerland); Aladdin (Topcon Europe, Visia Imaging, San Giovanni Valdarno, Arezzo, Italy); Pentacam HR (Oculus, Wetzlar, Germany); Galilei G4 (Ziemer Ophthalmic Systems AG, Port, Switzerland); IOLMaster 700 (Carl Zeiss Meditec, Dublin, CA, USA); Argos (Movu, Santa Clara, CA, USA); OA-2000 (Tomey, Nagoya, Japan); ANTERION (Heidelberg Engineering GmbH, Heidelberg, Germany).

## Data Availability

No new data were created or analyzed in this study. Data sharing is not applicable to this article.
